# How to manage the initiation of apomorphine therapy without antiemetic pretreatment: A review of the literature

**DOI:** 10.1016/j.prdoa.2022.100174

**Published:** 2022-12-19

**Authors:** Stuart H. Isaacson, Richard B. Dewey, Rajesh Pahwa, Daniel E. Kremens

**Affiliations:** a Parkinson’s Disease and Movement Disorders Center of Boca Raton, Boca Raton, FL, USA; b University of Kansas Medical Center, Kansas City, KS, USA; c Sidney Kimmel Medical College, Thomas Jefferson University, PA, USA

**Keywords:** Apomorphine, Titration, Trimethobenzamide, Nausea, Vomiting

## Abstract

**Introduction:**

Pretreatment with the antiemetic trimethobenzamide has been recommended practice in the United States (US) to address the risk of nausea and vomiting during initiation of apomorphine treatment. However, trimethobenzamide is no longer being manufactured in the US, and despite the recent update to the US prescribing information, there may be uncertainty regarding how to initiate apomorphine.

**Methods:**

To better understand why antiemetic pretreatment was recommended and if it is necessary when initiating apomorphine therapy, we performed a literature review of subcutaneous apomorphine therapy initiation with and without antiemetic pretreatment in patients with PD.

**Results:**

Three studies were identified as providing relevant information on antiemetic prophylaxis with initiation of injectable apomorphine. The first study demonstrated that nausea was significantly more common in patients who received 3-days of trimethobenzamide pretreatment compared with those who did not, while the primary endpoint of second study found no significant effect on the binary incidence of nausea and/or vomiting on Day 1 of apomorphine treatment. In the third study, which used a slow titration scheme for apomorphine, transient nausea was reported in just 23.1% of the antiemetic nonusers.

**Conclusions:**

Based on the reviewed trials and our clinical experience, we suggest that subcutaneous apomorphine therapy can be initiated using a slow titration scheme without antiemetic pretreatment.

## 1 Introduction

The US label for apomorphine subcutaneous injection (Apokyn®, Supernus Pharmaceuticals, Rockville, MD) has previously recommended starting antiemetic pretreatment with oral trimethobenzamide beginning three days prior to apomorphine initiation in patients with Parkinson’s Disease (PD) experiencing OFF episodes to reduce the risk for nausea and vomiting [1]. However, trimethobenzamide is no longer being manufactured in the US, and despite the recent update to the US prescribing information recognizing the ability to start apomorphine treatment at a lower dose (0.1 mL) *without* trimethobenzamide [1], there may be uncertainty regarding how to initiate this treatment. In the US, trimethobenzamide was the recommended antiemetic due to its use in the pivotal apomorphine trials [2], [3], [4]. Other antiemetics either worsen motor parkinsonism (e.g., metoclopramide, promethazine and prochlorperazine) [5], [6] or increase the risk of hypotension (e.g., ondansetron, granisetron, dolasetron, and palonosetron) [1], [7] and are therefore contraindicated. Domperidone is not approved for use in the US.

Pretreatment with trimethobenzamide beginning three days prior to the initial dose of apomorphine subcutaneous injection has been routinely used in the US; however, clinical experience with apomorphine suggests that prophylactic treatment is not always necessary nor clinically useful [8]. Indeed, antiemetics are not routinely used when beginning therapy with oral and transdermal dopamine agonists that are also associated with nausea during titration, and trimethobenzamide (a purported dopamine D2 receptor antagonist [9]) is not without adverse events in patients with PD, being associated with increased incidence of somnolence, dizziness, and falls. The American Geriatrics Society expert panel assessing potentially inappropriate medication for use in older adults (Beers criteria) cautions against the use of trimethobenzamide, suggesting that it is *“one of the least effective antiemetic drugs, yet it can cause extrapyramidal adverse effects”* [10]. To better understand why antiemetic pretreatment was recommended and if it is necessary when initiating apomorphine therapy, we reviewed studies of intermittent subcutaneous apomorphine therapy initiation with and without antiemetic pretreatment in patients with PD.

## 2 Literature review of clinical studies of apomorphine, with and without prophylactic antiemetic treatment

A broad literature review was performed using PubMed to identify studies (any design) that initiated subcutaneous apomorphine therapy with and without antiemetic pretreatment (mainly trimethobenzamide or domperidone). Key search terms included ‘subcutaneous’, ‘apomorphine’ and ‘Parkinson’s disease’ and the search was conducted between January 1985 and August 2022 in English. We also searched the reference lists of retrieved articles and contacted the authors for further information where needed.

Of the 498 reviewed references, just three studies were identified as providing relevant information on antiemetic prophylaxis with initiation of injectable apomorphine [11], [12], [13]. In the first study, Ondo et al. 2012 prospectively monitored 28 patients with idiopathic PD, receiving their first apomorphine injections to assess predictors of initial AEs and long-term tolerability [11]. Patients initiated apomorphine at a starting dose of 2 mg with subsequent titration if they failed to experience ON status; 11 patients were initiated following a three-day pretreatment with trimethobenzamide, while 17 patients were initiated following a single trimethobenzamide dose (n = 8) or no pretreatment (n = 9). Scoring of apomorphine-induced nausea was subsequently performed using a visual analogue scale within 40 min of the first injection to determine if trimethobenzamide helps prevent nausea.

The results demonstrated that nausea was significantly *more* common in patients who received 3-days of trimethobenzamide pretreatment compared with those who did not (RR: 3.44; 95 %CI: 0.93–12.74; p = 0.028). Other patient demographic variables (including prior history of nausea with other dopaminergics) did not predict the occurrence of nausea after apomorphine initiation. The authors concluded that *‘a three-day pretreatment dose of trimethobenzamide…does not reduce nausea’.*

Hauser et al. 2014 [12] conducted a randomized, placebo-controlled trial to better understand whether trimethobenzamide could reduce the incidence of nausea, vomiting, and discontinuation rates due to nausea and vomiting at different time periods following initiation of apomorphine. Patients (N = 182) were randomized 3:1 to receive trimethobenzamide 300 mg, three times daily or matching placebo beginning 3 days before initiation of subcutaneous apomorphine injections. Apomorphine was titrated from a starting dose of 2 mg to clinical response (up to 6 mg). In a phased withdrawal design, following 4 weeks of therapy, patients initially assigned to trimethobenzamide were re-randomized 2:1 to continue trimethobenzamide or switch to placebo for the next 4 weeks of treatment. At this time, patients still assigned to trimethobenzamide were again re-randomized (1:1) to either continue trimethobenzamide or switch to placebo for a third 4-week period.

The study found no significant effect on the binary incidence of nausea and/or vomiting on Day 1 of apomorphine treatment (primary endpoint: 16.2 % with trimethobenzamide vs 22.7 % with placebo; p = 0.09) [12]. While there was a significantly lower incidence of nausea and/or vomiting with trimethobenzamide for the first 2 months of the 3-month study (36.9 % vs 54.5 % in the first 4-weeks and 23.8 vs 46.7 % in the second 4-weeks, respectively), there was no difference during month 3 (33.3 % in each group) and the incidence of discontinuation due to these AEs was slightly higher with trimethobenzamide (3.7 % vs 1.9 % placebo). Of note, dopamine agonist use at baseline appeared to have a greater influence on emergence of nausea and vomiting with apomorphine initiation than trimethobenzamide use. Based on *post hoc* analysis, significantly fewer patients taking dopamine agonists experienced nausea and/or vomiting than those not taking dopamine agonists (40.2 % vs 67.2 %; p less than 0.0052, respectively). Moreover, when controlling for dopamine agonist use, there was no significant difference for trimethobenzamide in any study period [12].

Of note, in this study predosing with trimethobenzamide was associated with a lower percentage of patients experiencing a “full” ON response on Day 1. The percentage of patients who turned fully ON (trimethobenzamide vs placebo pretreatment) was 65.4 % vs 72.7 % (p = 0.17) following the first apomorphine (2 mg) injection and 74.5 % vs 90.0 % (p = 0.008) following the second injection (2–4 mg) [12]. Trimethobenzamide use also appeared to affect onset latency; the median time to ON following the first apomorphine (2 mg) dose was 25.0 vs 20.0 min (trimethobenzamide vs placebo) and following the second (2–4 mg) dose was 20.0 vs 16.5 min. Taken together, the results from the study indicate that prophylactic trimethobenzamide had little meaningful effect on nausea or vomiting following initial apomorphine titration (doses up to 4–5 mg) and may slightly reduce the probability of turning ON as well as delay the time to ON.

In the third study reported by Hattori et al. 2014 [13], 31 patients with PD and motor fluctuations were included in a 3-month trial consisting of a titration phase where patients were titrated to their optimal maintenance dose, followed by a 12-week open-label phase, and ending with pre- and post-dose assessment of apomorphine or placebo injection using a randomized, double-blind, placebo-controlled crossover design. Because there was no clear evidence showing the necessity of antiemetic pretreatment when initiating apomorphine, prophylactic antiemetic use was prohibited unless the patient had already been receiving antiemetic treatment prior to study start. Five patients initiated apomorphine (starting at 1 mg and increasing in 1 mg increments) with a concomitant antiemetic (domperidone or mosapride citrate), and the remaining 26 without antiemetic use. Nausea was reported in one (20 %) of the antiemetic users and 6 (23.1 %) of the antiemetic nonusers, 3 of whom required active management (adding domperidone n = 2, reducing apomorphine dose n = 1) with nausea disappearing soon afterwards. None of the remaining 20 antiemetic nonusers reported any gastrointestinal upset. It is also pertinent to note that 87 % of study patients were using other dopamine agonists at baseline, and the mean maintenance dose per apomorphine injection was 2.6 mg which is lower than that used in most Western studies.

## 3 Practical initiation of apomorphine therapy without anti-emetic pretreatment

Because trimethobenzamide is scarcely available in the US and domperidone is not an FDA-approved therapy, treating physicians need to find alternative strategies to initiate apomorphine therapy. Based on the reviewed trials and our clinical experience, we suggest that subcutaneous apomorphine therapy can be initiated without antiemetic pretreatment. Patients who are believed to be at higher risk of developing nausea and/or vomiting can initiate treatment with a lower 1 mg dose. After the initial dose, apomorphine can then be titrated to identify an optimal dose based on therapeutic response and tolerability. For example, if the patient experiences little benefit but no adverse effects with the initial 1 mg dose, the dose can then be increased to 2 or 3 mg. If the initial dose causes mild nausea, an increase to 1.5 or 2 mg can be attempted; if nausea was more than mild with the initial dose, then the initial dose can be repeated for 2–7 days to provide time for tolerance to develop. If severe nausea and/or vomiting occurs with the initial dose, then the dose can be cut in half, and repeated for 2–7 days until tolerance develops, followed by dose increases as tolerated. Using a ‘start low and go slow’ flexible titration strategy is similar to what was used in the Hattori et al. study [13], where incidence of nausea was ∼ 20 %, was mild in severity, and did not result in apomorphine discontinuation.

In our own practice, we have used a similar clinical strategy for initiating apomorphine as we have used for other oral- or transdermally- administered dopamine agonists. Beginning with a low dose and titrating flexibly based on tolerability and efficacy has allowed patients to reach apomorphine doses optimized to achieve a rapid onset (within 15 min), robust response (similar to their levodopa best ON), and reliable effect (most doses), without dose-limiting adverse effects (i.e., nausea). Our clinical experience is supported by recent data from an evaluation of almost 2000 patients initiating apomorphine subcutaneous injection in the US since trimethobenzamide became scarce. In a review of the Apomorphine Clinical Educator database that is sponsored by Supernus Pharmaceuticals, the percentage of patients initiating apomorphine without trimethobenzamide pretreatment rose from less than 35 % (in 2019) to over 85 % (in 2021). Yet the percentage of patients continuing apomorphine therapy for at least 3 months remained stable [14].

While the scarcity of available antiemetic in the US led us to reconsider how to initiate and titrate apomorphine to achieve an optimal clinical dose, our observations may also be helpful outside the US. Although our described clinical experience and the reviewed trials were with intermittent subcutaneous injection of apomorphine, the same clinical approach may also be applicable to the use of continuous subcutaneous apomorphine infusion (CSAI) which is under development in the U.S.

## Declaration of Competing Interest

The authors declare that they have no known competing financial interests or personal relationships that could have appeared to influence the work reported in this paper.
